# Spontaneous Thrombosis in Giant Aneurysm of the Anterior Communicating Artery Complex in Pediatric Age: Five-Year Follow-Up

**DOI:** 10.1155/2014/236041

**Published:** 2014-08-28

**Authors:** Vítor M. Gonçalves, N. Cristino, M. Cunha e Sá

**Affiliations:** Neurosurgery Department, Garcia de Orta Hospital, Avenida Torrado da Silva, 2801-951 Almada, Portugal

## Abstract

Intracranial aneurysms are rare in the pediatric population, especially in infancy, representing less than 1% of all aneurysms. In this age group, they are more frequent at the carotid bifurcation and in the posterior circulation, with a greater number of giant aneurysms and spontaneous aneurysm thrombosis when compared with the adults. They are life-threatening, and, therefore, early investigation, characterization of the lesion, and treatment are essential. The appropriate management depends on the child's condition, aneurysm characteristics, and the experience of a multidisciplinary team. Noninvasive and radiation-free imagiological studies play an important role in the diagnosis and follow-up of these young patients. We present the case of a 3-month-old boy with an intracranial hemorrhage secondary to the rupture of a giant aneurysm of the anterior communicating artery complex, with spontaneous thrombosis, which is a rare situation due to its location. A conservative approach was assumed and noninvasive evolutive imagiological studies revealed a reduction in the thrombosed aneurysm size and no signs of recanalization. The child recovered to his baseline neurological condition and has had no rehemorrhage until 5 years of follow-up.

## 1. Introduction

The incidence of intracranial aneurysms in childhood is rare, especially in infancy [1, 2], with a reported prevalence ranging from 0,5 to 4,6% among all intracranial aneurysms [3]. Differently from adults they occur more often in boys than in girls (1,4 : 1) [3]. In childhood aneurysms are more frequent at the carotid bifurcation [4–6] and in the posterior circulation [6, 7]. Often they are large (10–25 mm) and 16 to 54% are giant (>25 mm) [6]. Multiple aneurysms are less frequent in children than in adults, with an incidence of 6,4% in pediatric cases [6].

Spontaneous thrombosis (partial or complete) occurs between 8,3 [3] and 16,9% [5] of all pediatric intracranial aneurysm patients, which is more frequent than in adults. Complete thrombosis is very unusual, reported in 1 to 16% of cases [8]. Even in the aneurysms which are considered “completely thrombosed,” the neck of the aneurysm is usually seen on angiography [8]. The etiology and pathogenesis of pediatric intracranial aneurysms are still unknown [3]. Whether congenital [7] or not [9], they are probably the result of an interplay between structural changes in the vessel wall and hemodynamic stress [3, 10]. Traumatic and infectious diseases also contribute to aneurysms in the pediatric population [3].

The presenting features of intracranial aneurysms in the pediatric population are different from those in adults. The incidence of subarachnoid hemorrhage in previous reports varies from 35 to 100% [3, 5, 11], most of them having good Hunt-Hess degrees [12]. In pediatric patients the outcome is similar or slightly better than in adults [13].

We present the case of a young boy with an intracranial hemorrhage caused by the rupture of an anterior communicating artery complex giant aneurysm with spontaneous thrombosis, which is a rare situation owing to its location.

## 2. Case Presentation

**Figure 1:**
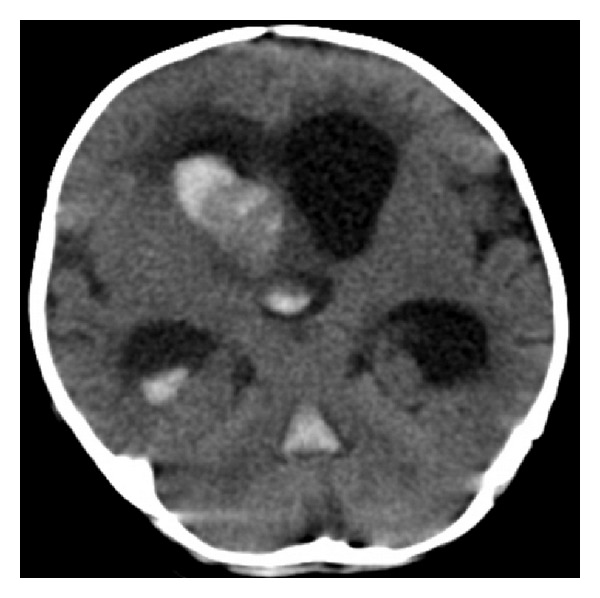
Cranial preoperative CT-scan: tetraventricular hemorrhage (Fisher 4) and acute hydrocephalus.

A 3-month-old boy, delivered in eutocic birth, with good height-weight evolution and irrelevant personal and familial past diseases, presented with a complex partial seizure, preceded by a loud scream and ocular infraversion. Spontaneous recovery occurred in few minutes. The patient was admitted in a pediatric emergency room. He was apyretic, hemodynamically stable, eupneic with good peripheral oxygen saturations, flat, and pulsating fontanelle. He had normal blood and urinary laboratory tests. No signs of traumatic brain injury were seen. Fifteen minutes after admission, he suffered another seizure. A postictal period with no reaction to pain, bradycardia, and a hard and bulging fontanelle was noticed. A cranial computed tomography (CT) scan was performed (Figure 1).

The patient was transferred to a pediatric intensive care unit, and treatment with phenytoin 15 mg 12/12 h, mannitol, sedoanalgesia with midazolam, and fentanyl was started. He was intubated and connected to mechanical ventilation. An external ventricular drain was urgently placed through the left external angle of the anterior fontanelle.

**Figure 2:**
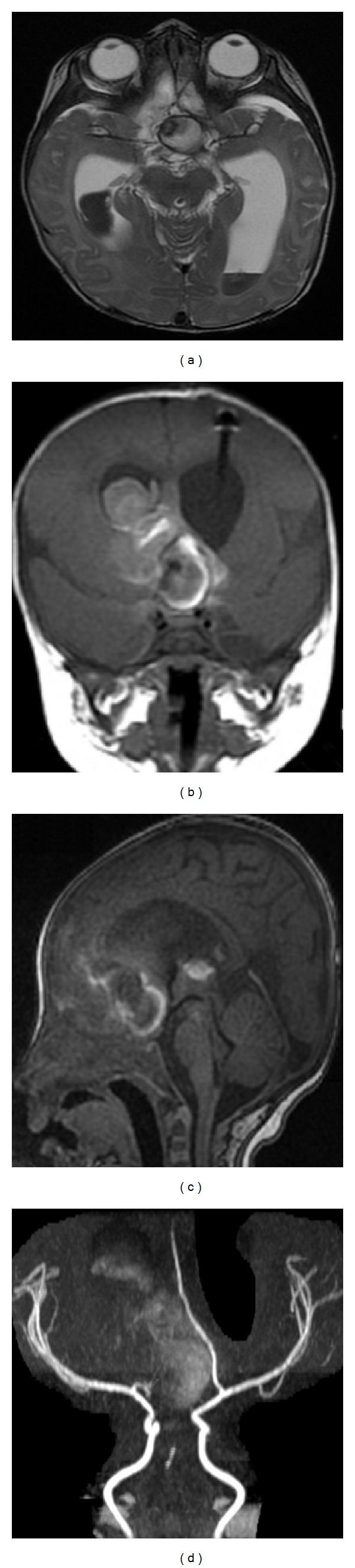
((a), (b), and (c)) Early stage MRI demonstrating intraventricular hemorrhage with associated hydrocephalus and a large extra-axial suprasellar lesion measuring 26 mm diameter with a slight filling and an endoluminal thrombus; (d) on MRA no lesion is visualized, likely due to its partial thrombosis and sluggish blood flow. Only its repercussion on middle caliber vessels can be appreciated: left A1 segment of the anterior cerebral artery superiorly and laterally deviated and right A1 only visible on its proximal segment.

A magnetic resonance imaging (MRI) and angiography (MRA) were performed in the first 24 hours after the hemorrhage (Figure 2). A suprasellar lesion with 26 mm diameter compatible with a giant aneurysm of the anterior communicating artery complex was documented. A slight filling of the suspected aneurysm and an endoluminal thrombus were visualized.

**Figure 3:**
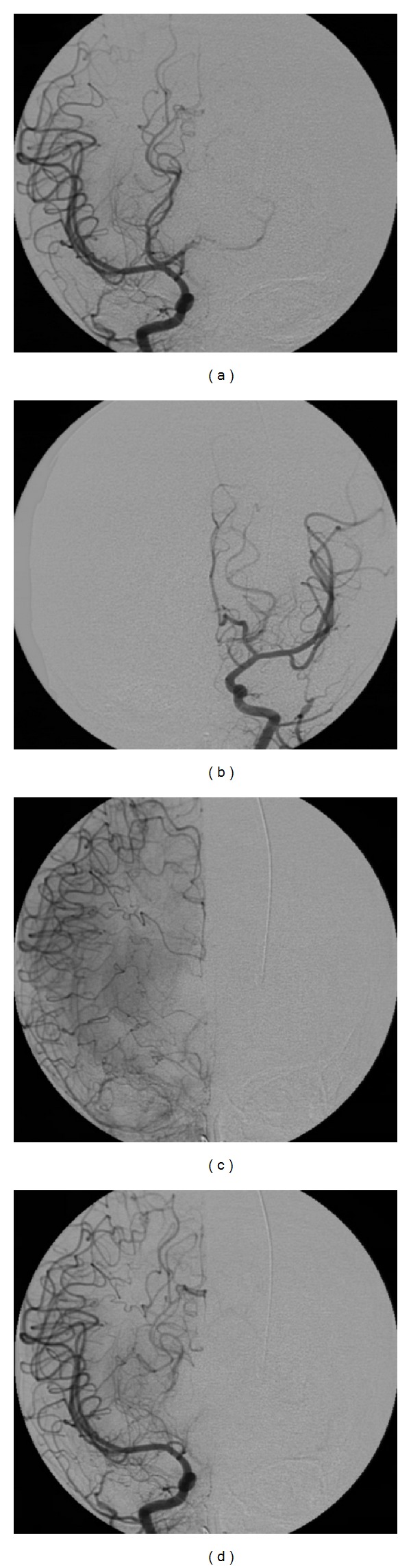
(a) Right internal carotid arteriogram: visualization of the A1 proximal segment. No filling of the A2 segment or the aneurysm. (b) Left internal carotid arteriogram: A1 superiorly and laterally deviated. ((c) and (d)) Right internal carotid arteriogram: right ACA territory vascularization due to leptomeningeal anastomoses between right MCA and PCA.

A diagnostic cerebral angiography (Figure 3) was done three days after MRI and MRA. No therapeutic endovascular procedure was performed once there was no filling of the suspected aneurysm, at this point. A spontaneous thrombosis was assumed to have occurred. No other lesions were identified.

These features were considered to be consistent with an intracranial hemorrhage secondary to the rupture of an anterior communicating artery complex giant aneurysm, with subsequent spontaneous thrombosis.

On the 5th day of treatment, assisted ventilation and sedation were suspended and the patient was extubated. A CT scan was repeated 11 days after the initial hemorrhage, which demonstrated persistence of the hydrocephalus. A medium-low pressure ventriculoperitoneal shunt was surgically inserted at that time.

**Figure 4:**
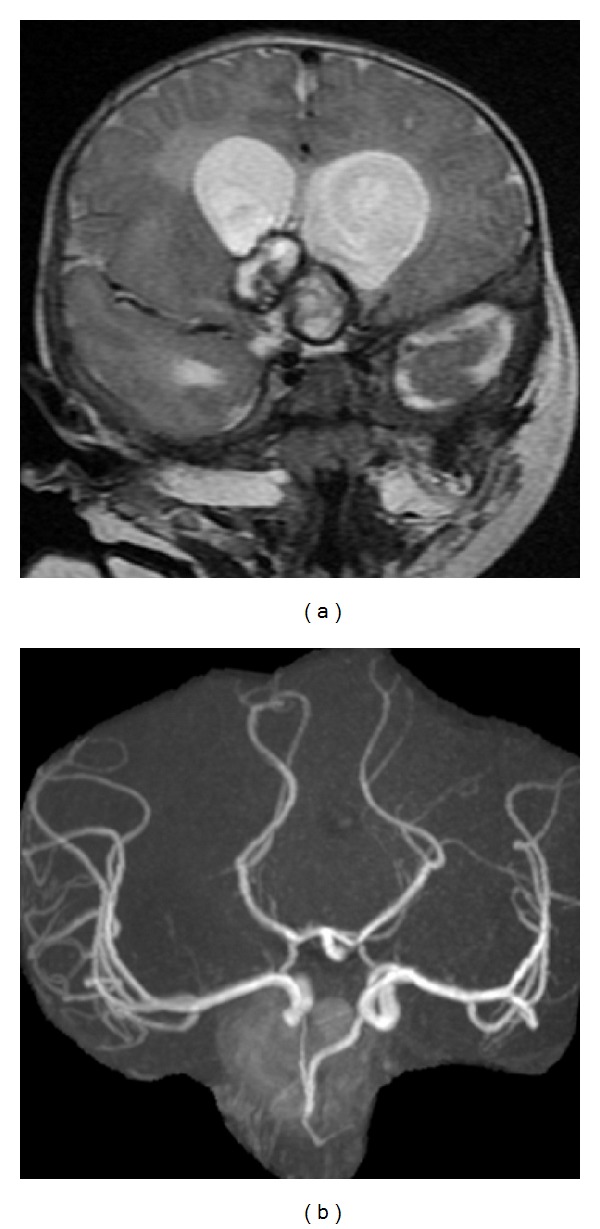
MRA performed 2 weeks after onset of hemorrhagic event. (a) No active hydrocephalus and volume reduction of the thrombosed aneurysm; (b) right ACA visualization.

With two weeks of treatment the electroencephalogram reported low paroxysmal activity, bilaterally, on the parietal region. Progressive phenytoin suspension and its substitution for carbamazepine were endeavored. The MRA was repeated at this point (Figure 4).

At this early stage a clinically significant improvement was achieved. The child recovered to his baseline neurological condition and had no more seizures. After 26 days of hospitalization, he was discharged from hospital, without neurological deficits.

**Figure 5:**
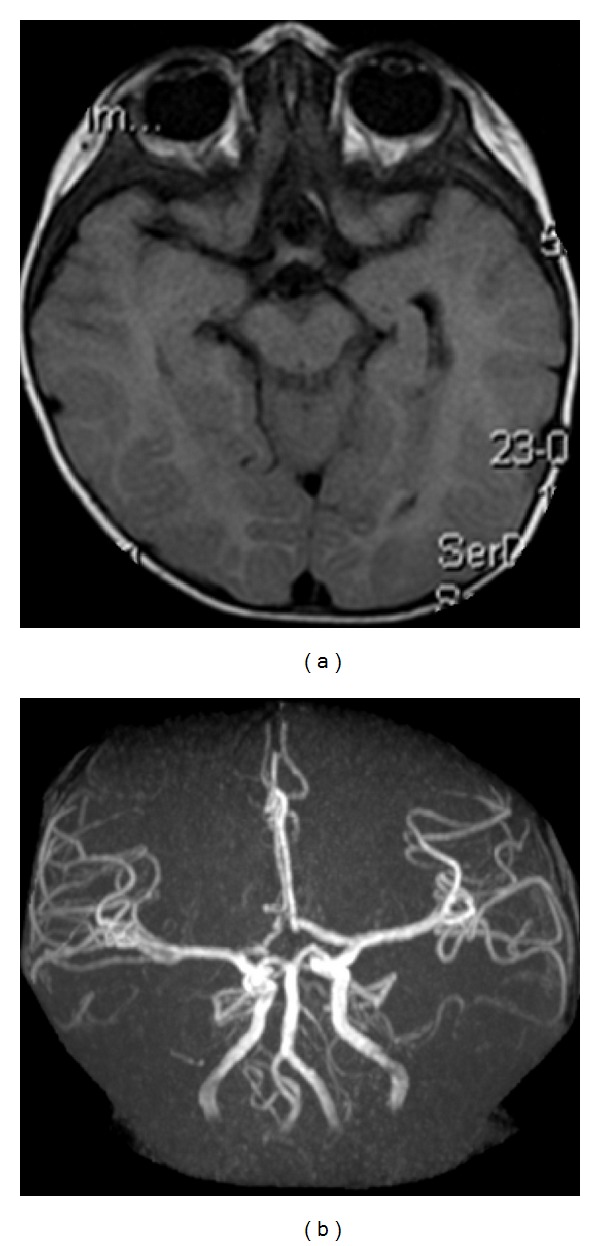
MRA performed 3 months after the initial hemorrhagic event. (a) Considerable volume reduction of the thrombosed aneurysm; (b) patent right ACA. No residual aneurysmal neck was documented.

The patient was followed up in neuropediatric and pediatric neurosurgery outpatient clinic. He totally recovered and repeated the MRA when he was 6-month old, which disclosed further volume reduction of the thrombosed aneurysm and good cerebral vascularization (Figure 5). No neurological symptoms due to mass effect were detected.

**Figure 6:**
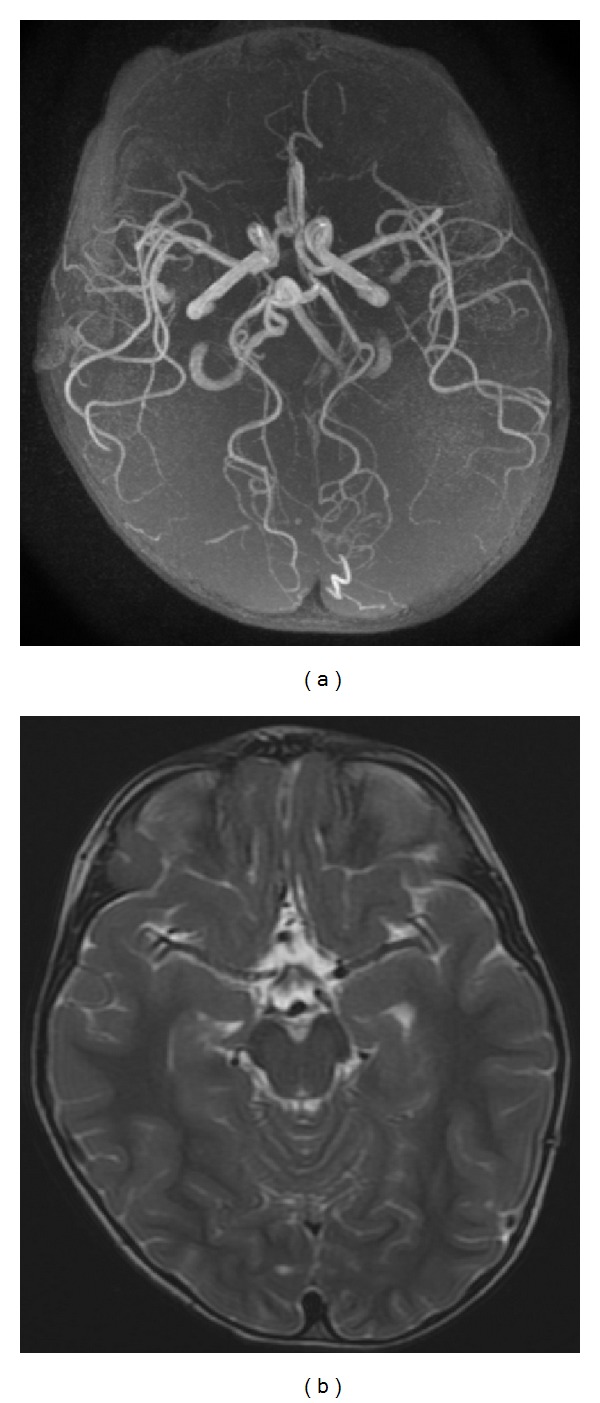
((a) and (b)) Five-year follow-up MRA demonstrating no recanalization of the giant anterior communicating artery complex aneurysm and a permeable right ACA.

Further angiographic studies were performed at 6 and 12 months and subsequently with yearly intervals until 5 years after aneurysm rupture. The last MRA showed no recanalization of the anterior communicating artery aneurysm and a permeable right ACA (Figure 6). At five-year follow-up, the child remains neurologically intact, living without restrictions. Seizures ceased, allowing the suspension of the prescribed medical therapy.

## 3. Discussion

Even though intracranial aneurysms are rare in the pediatric population, they have high morbidity and mortality, and, therefore, early investigation, characterization of the lesion, and treatment are essential. The aneurysms located in the anterior communicating artery complex are relatively rare in children, contrasting to what happens in the adult population, where they are the most frequent. The appropriate management depends on the child's condition, aneurysm characteristics, and the experience of the multidisciplinary team, including neurosurgeons and neuroradiologists. The imagiological studies include digital angiography, CT angiography, and magnetic resonance angiography. The last two are noninvasive studies and assume an important role on the diagnosis and follow-up of these patients [14]. Ionizing radiation is an established risk factor for the development of neoplastic diseases [15–19], and children may be especially predisposed to its harmful effects [17, 20–24]. The authors consider that pediatric patients requiring multiple CTA scans for the follow-up of spontaneous thrombosed aneurysms represent a high risk group for radiation-induced diseases. For this reason performing MRA for surveillance imaging is encouraged [25], although it is important, during the evolutive control of the aneurysm, to perform a digital subtraction angiography to check if the aneurysm has recurred [3, 26]. In the present case, the spontaneous aneurysmal thrombosis allowed us to assume a conservative management. It was a clinical situation with obvious technical difficulties, both microsurgical and endovascular. The evolutive follow-up with noninvasive imagiological studies revealed a volume reduction of the thrombosed aneurysm.

Further clinical and imagiological follow-up is needed once recanalization of thrombosed lesions, although rare [27, 28], cannot be anticipated [14]. Delayed recanalizations have been described [29]. The timing and frequency of subsequent cerebrovascular imaging and treatment are still on debate [29]. Some authors advocate performing control angiographic studies at 3, 6, and 12 months and subsequently with yearly intervals until at least 3-year follow-up, due to the risk of recurrence and rehemorrhage [3]. In this particular case, the child remains neurologically intact and having a normal life, without imagiological evidence of recanalization at 5-year follow-up. However, further reports and clinical series are necessary to assess current outcome indicators and to clarify and better understand the natural history of this life-threatening disease.

## 4. Conclusion

Pediatric intracranial aneurysms are rare but associated with high morbidity and mortality, demanding an early diagnosis, characterization of the aneurysm, and delineation of an effective treatment strategy. Natural history of completely thrombosed aneurysms is still uncertain, ranging from benign to the immediately life-threatening. The appropriate management depends on the child's condition, aneurysm characteristics, and the experience of the multidisciplinary team, including neurosurgeons and neuroradiologists. Noninvasive and radiation-free imagiological studies assume an important role in the diagnosis and follow-up of these young patients.

## Abbreviations

A1: A1 segment of the anterior cerebral artery
A2: A2 segment of the anterior cerebral artery
ACA: Anterior cerebral artery
MCA: Middle cerebral artery
PCA: Posterior cerebral artery
CT: Computed tomography
MRI: Magnetic resonance imaging
MRA: Magnetic resonance angiography.
